# Adaptation and Retention of Digitally Fabricated Presurgical Orthopedic Plates for Infants with Cleft Lip and Palate

**DOI:** 10.3390/dj14100665

**Published:** 2026-10-12

**Authors:** Fatima Shakir, Mushriq Abid

**Affiliations:** Department of Orthodontic, College of Dentistry, University of Baghdad, Baghdad 10001, Iraq

**Keywords:** accuracy, fitness, cleft lip and palate, PETG, direct printed plates, presurgical orthopedics, PETG/TPU

## Abstract

**Objectives:** This study aimed to evaluate the fitness and retention of presurgical infant orthopedic (PSIO) plates fabricated from three materials—polyethylene terephthalate glycol-modified (PETG), PETG/polyurethane (PETG/TPU), and direct-printed Tera Harz resin—within a standardized in vitro framework. **Methods:** Thirty molding plates (ten per material) were fabricated on stereolithographic models. Fitness was assessed virtually using Exocad software DentalCAD 3.3 SR1 (exocad GmbH, Darmstadt, Germany), with gap distances measured at 36 standardized points across anterior, middle, and posterior regions. Statistical analysis was performed using Welch’s ANOVA and Games–Howell post hoc comparisons. Retention was tested on soft gum casts using a digital force gauge under simulated intraoral conditions. Plates were seated with denture adhesive, pressurized, and subjected to perpendicular traction until detachment. Mean retentive force was calculated from three repeated trials per plate. **Results:** PETG plates demonstrated the closest adaptation (0.226 ± 0.060 mm), significantly outperforming direct-printed plates (0.386 ± 0.120 mm; *p* = 0.006). PETG/TPU plates showed intermediate values (0.321 ± 0.134 mm) without significant deviation from either group. Region-specific analysis revealed the greatest misfit anteriorly, with PETG consistently achieving superior conformity. PETG exhibited the highest mean retentive force (4.30 N), significantly greater than PETG/TPU (2.28 N) and direct-printed plates (0.93 N; *p* < 0.001). PETG/TPU also performed significantly better than direct-printed plates. **Conclusions:** Material choice critically influences both the fit and retention of PSIO plates. PETG demonstrated more favorable adaptation and retention under controlled experimental conditions, suggesting potential advantages for appliance stability in presurgical infant orthopedic applications, while PETG/TPU offered moderate performance and direct-printed plates showed the weakest outcomes. Nonetheless, claims of clinical reliability or material superiority cannot be substantiated without further validation. Comprehensive longitudinal clinical studies are therefore required to confirm these preliminary observations and establish their relevance in patient care.

## 1. Introduction

Cleft lip (CL), cleft palate (CP), and cleft lip and palate (CLP) are among the most common congenital craniofacial anomalies affecting the oral cavity and lips [1,2]. Infants with cleft lip and/or palate often experience feeding difficulties, including nasal regurgitation, inability to form an effective latch, increased effort during feeding, and early fatigue, all of which can compromise adequate nutrition if not properly managed [2,3,4,5,6].

The introduction of intraoral scanning, CAD/CAM technology, and additive manufacturing has transformed the fabrication of presurgical orthopedic appliances [7]. Digital workflows eliminate many of the limitations associated with conventional impressions while enabling rapid production of individualized appliances with improved reproducibility and reduced laboratory time [8,9,10,11]. Thermoformed aligner materials such as PETG and multilayer PETG/TPU, together with directly printable photopolymer resins, have recently expanded the range of available manufacturing options [12,13,14,15]. These innovations streamline workflows by replacing labor-intensive manual procedures with digital solutions, allowing the production of customized appliances at the point of care [16,17,18]. 

Recent investigations have demonstrated the feasibility of digitally fabricated PSIO appliances, including dual-layered aligners and directly printed nasoalveolar molding devices. These studies reported promising clinical outcomes and improved workflow efficiency; however, they primarily focused on treatment feasibility rather than the comparative biomechanical performance of different fabrication materials [19,20,21,22,23].

Retention and fitness of presurgical plates are not merely technical considerations but central determinants of treatment success in infants with cleft lip and palate. A well-adapted appliance ensures stability within the oral cavity, promotes proper maxillary growth, facilitates feeding, and enhances parental compliance. Conversely, inadequate retention or poor fit can compromise arch development, reduce the effectiveness of nasoalveolar molding, and ultimately affect surgical outcomes [24,25,26]. 

Although digital technologies such as CAD/CAM design and 3D printing have introduced remarkable precision and efficiency into cleft care, these advances cannot replace the fundamental requirement that the appliance must remain securely in place and conform accurately to the infant’s anatomy [7,21,27,28,29,30]. The clinical promise of digital innovation will only be realized if retention and fit are rigorously evaluated and optimized. To the best of our knowledge, no previous investigation has systematically compared thermoformed PETG, dual-layer PETG/TPU, and directly printed Tera Harz resin PSIO appliances using standardized assessments of both adaptation accuracy and retentive force. Therefore, the present study aimed to compare the fitness and retention of these three materials within a standardized digital in vitro framework. We hypothesized that significant differences would exist among the tested materials with respect to both adaptation accuracy and retention. The findings of this in vitro study may provide preliminary evidence regarding the fitness and retention characteristics of the proposed material, which could inform future improvements in presurgical appliances for cleft lip and palate management.

## 2. Materials and Methods

### 2.1. Study Design

This in vitro experimental study compared the adaptation accuracy and retention of presurgical infant orthopedic (PSIO) plates fabricated from three contemporary materials using a standardized digital workflow.

### 2.2. Ethical Approval

Informed consent was obtained from the parents of the participating child, and the study was approved and conducted in accordance with the Declaration of Helsinki by the Ethical Committee of the College of Dentistry, University of Baghdad (Reference Number: 1104; Approval Date: 20 November 2025).

### 2.3. Sample Size Calculation

Prior to the main experiment, a pilot study was conducted to estimate the expected differences among the investigated materials. The pilot data demonstrated mean fitness values of 0.146 ± 0.012 mm for PETG, 0.250 ± 0.073 mm for TPU/PETG, and 0.294 ± 0.113 mm for the directly printed Tera Harz resin group. The effect size (Cohen’s *f*) was calculated from the pilot study by determining the variance of the group means relative to the pooled within-group variance. Based on these pilot measurements, a large effect size (*f* ≈ 0.80) was obtained. Subsequently, an a priori power analysis was performed using G*Power software 3.1.9.7 (Heinrich Heine Universität Düsseldorf, Germany) for a one-way ANOVA with three independent groups, a significance level (α) of 0.05, and a statistical power of 80%. The analysis indicated that a minimum total sample size of approximately 21 specimens was required. To enhance the robustness of the findings and account for potential experimental variability, 30 specimens were ultimately included in the study, corresponding to 10 specimens per group. Data were processed using SPSS Statistics version 25 (IBM, Armonk, NY, USA).

### 2.4. Fabrication of Individual Orthopedic Appliances

An impression was obtained from an infant presenting with unilateral cleft lip and palate (CLP) using a fast-setting polyvinylsiloxane impression material (Prestige, Florence, Italy) in a custom-fabricated pediatric acrylic tray. The procedure was performed with the infant fully awake and positioned in an almost upright orientation to minimize airway risk and ensure patient safety.

The impression was subsequently poured with Type IV dental stone (Zhermack, Badia Polesine, Italy). The resultant stone cast was digitized using an E1 optical extraoral scanner (3Shape TRIOS, Copenhagen, Denmark). The scan data were stored in standard tessellation language (STL) format and imported into Exocad in-lab exocad DentalCAD 3.3 for further processing. To facilitate accurate model fabrication, the pronounced undercut in the cleft region was virtually blocked prior to printing. The materials used in this study are listed in Table 1.

Digital data were exported for stereolithographic (SLA) printing. Ten study models were fabricated for plate construction, and an additional model was printed with a 2 mm soft gum layer to evaluate retention (Asiga Dental Model and Asiga Dental Gum Resin, Sydney, NSW, Australia).

Thirty molding plates were fabricated on printed models. Three different materials were employed, with ten plates fabricated from each. Plates were manufactured from polyethylene terephthalate glycol-modified (PETG; Erkodur, Erkodent, Pfalzgrafenweiler, Germany); another ten plates were produced from double-layer sheets composed of a hard copolyester (PETG) surface and a soft polyurethane (TPU) lining (Erkoloc-pro, Erkodent, Pfalzgrafenweiler, Germany); and ten plates were prepared from direct-printed material of Tera Hartz resin. For each printed model, one plate was prepared from each of the three materials.

The thermoforming sheets utilized in this study included Erkodur and Erkoloc-pro, both manufactured by Erkodent Erich Kopp GmbH (Pfalzgrafenweiler, Germany). Erkodur was employed in the form of clear PET-G sheets with a thickness of 1.00 mm and dimensions of 125 × 125 mm and used in a single-layer configuration for the fabrication of rigid appliances such as aligners and retainers. In contrast, Erkoloc-pro consisted of a dual-layer structure combining an outer PET-G surface with an inner thermoplastic polyurethane (TPU) lining; in this study, transparent sheets of 1.00 mm thickness and 125 × 125 mm dimensions were used. The bi-layer design of Erkoloc-pro is intended to provide rigidity externally while offering internal resilience and patient comfort.

All materials used for plate construction were approved by the U.S. Food and Drug Administration (FDA). A vacuum-forming machine (Biostar Thermoforming Unit, Scheu Dental, Iserlohn, Germany) was employed to fabricate aligners from PETG and PETG/TPU sheets. The sheets had an initial thickness of 1 mm, which was reduced to 0.8 mm following the thermoforming process. Following fabrication, the plates were carefully removed from the models to minimize the need for trimming and to prevent breakage. Any irregular borders or ridges were subsequently refined using handpiece burs to ensure smooth finishing. All orthopedic plates were fabricated using a standardized design and a uniform nominal thickness of 0.8 mm to minimize manufacturing variability among specimens. Although these standardized fabrication conditions promoted geometric consistency, plate mass was not directly measured and may have varied among materials due to differences in material density.

Direct-printed aligner plates were fabricated using Tera Harz resin with a SprintRay digital light processing (DLP) stereolithography 3D printing system. Digital impressions of the patient’s cast were obtained using an optical scanner and exported as STL files, which were subsequently imported into orthodontic CAD software to design the aligner geometry. The finalized designs were prepared in SprintRay’s print management software, with orientation set flat to minimize warping and supports configured at standardized density and contact size to ensure stability during fabrication. Printing was performed at a standardized final thickness of 0.8 mm using Tera Harz resin. Following fabrication, the aligners were subjected to a standardized post-processing protocol that included rinsing twice in isopropyl alcohol (2 × 5 min) to remove residual resin, air-drying, and ultraviolet light curing in a dedicated curing unit for 20 min at 60 °C to achieve complete polymerization and mechanical stability. Supports were then carefully removed, and the aligners were polished and finished to eliminate surface irregularities and ensure clinically acceptable smoothness. All parameters—including printer model, printing technology, plate thickness, orientation, support configuration, washing duration, curing time and temperature, and finishing procedures—were standardized to maximize reproducibility of the direct-printing protocol. All plates were transparent, while the directly printed plates exhibited a slightly foggy appearance compared to the others.

### 2.5. Data Acquisition and Measurement

#### 2.5.1. The Scanning Processes

All samples were digitized using an E1 optical extraoral scanner (accuracy: 10 μm), which was calibrated prior to each scanning session. Each sample was positioned and stabilized on the scanning platform with modeling clay to ensure immobility during acquisition. The palatal surface was oriented parallel to the camera module, thereby defining the region of interest. 

To enhance scan fidelity, an anti-glare spray and titanium oxide powder (Dentaco, Essen, Germany) were applied to the surfaces. The powder was administered twice from different directions to optimize light diffusion and minimize reflection artifacts. Subsequently, the three-dimensional parameters of the tissue surface and the corresponding digital models were acquired individually. Following this, each molding plate was placed on its respective model and scanned in combination to obtain integrated datasets.

#### 2.5.2. Virtual Matching Process

Virtual alignments were performed using Exocad software. The procedure was based on three STL scan files generated from the cast, the molding plate, and the cast–plate combination. These files were sequentially imported into Exocad. The combined scan of the plate and cast served as the alignment guide, enabling precise positioning of the individual files.

**Figure 1:**
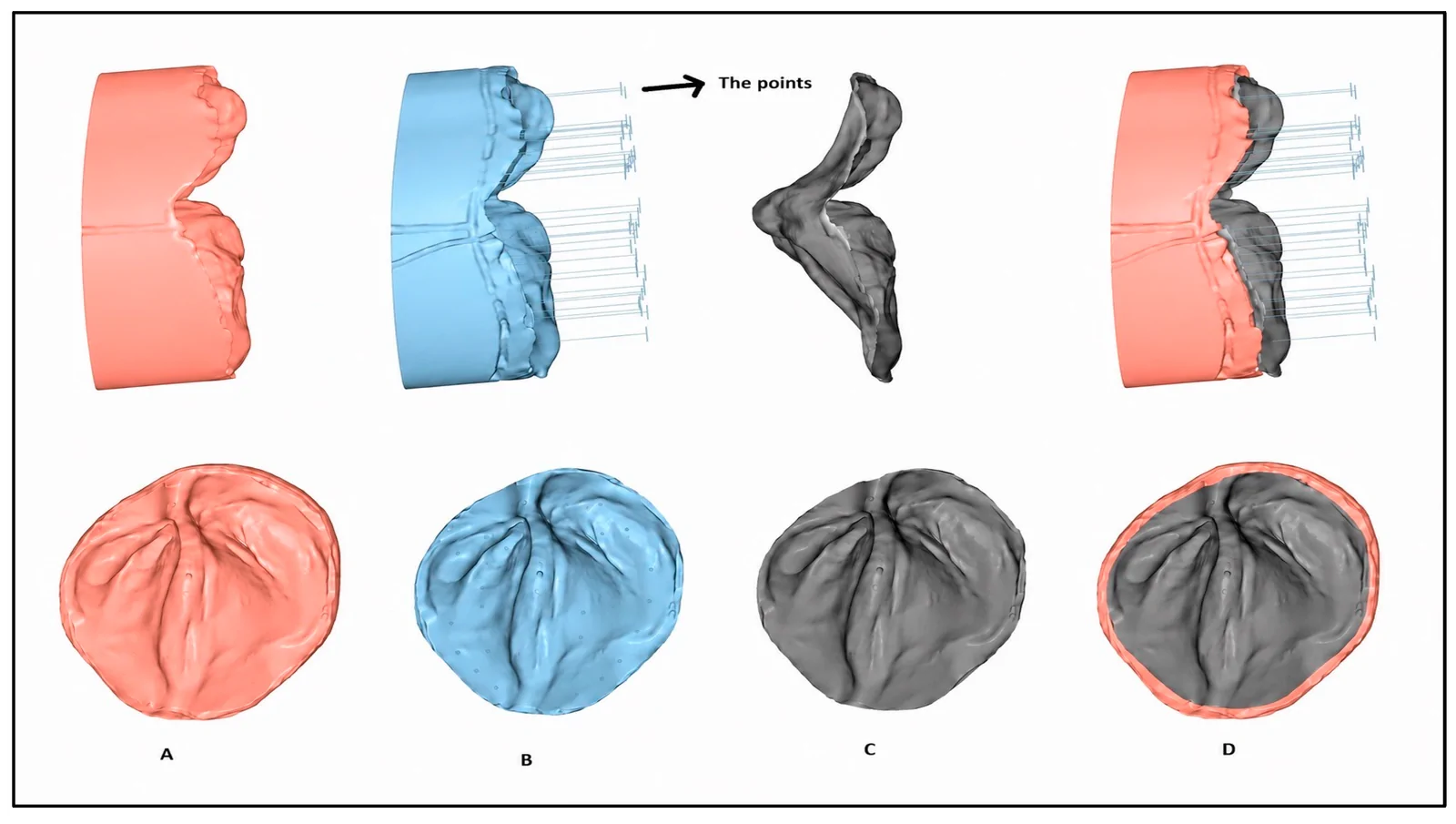
Virtual setup showing the alignment process: (**A**) first, the cast was scanned into Exocad software; (**B**) the alignment guide was scanned into Exocad; (**C**) the plate was scanned and aligned with the cast and the alignment guide; (**D**) the scanned components were merged together, and finally, the alignment guide was concealed, while the cast and plate remained aligned for analysis.

The alignment procedure followed a stepwise configuration within Exocad. First, the cast was scanned into Exocad software, followed by digitization of the alignment guide. The plate was subsequently scanned and aligned with both the cast and the alignment guide. Thereafter, all scanned components were merged, and the alignment guide was concealed, leaving the cast and plate in proper alignment for subsequent analysis (Figure 1).

#### 2.5.3. Measurement Process

The measurement procedure was conducted virtually on the original STL file. A point guide was generated to standardize the location of each measurement point across all materials, ensuring consistency. The guide consisted of 36 points, systematically distributed into three regions: anterior, middle, and posterior. Each region contained 12 points, with six positioned on the right side and six on the left side.

**Figure 2:**
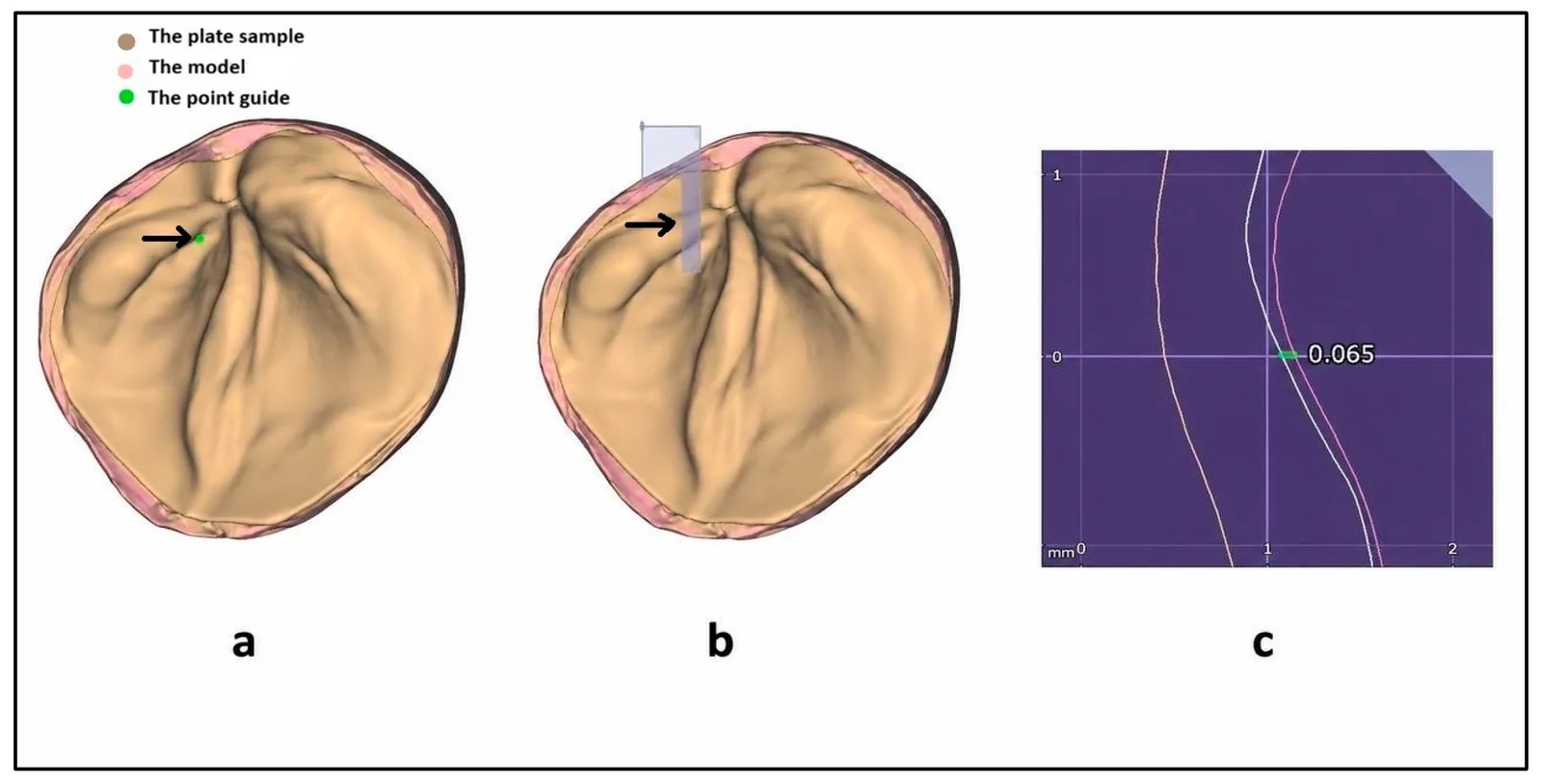
Measurement procedure applied in this study: (**a**) magnified visualization of an individual reference point (arrowed); (**b**) a sector drawn perpendicular through the designated reference points (indicated by arrows); (**c**) a sector intersecting the cast (the blue line), the molding plate (the pink line), and the guiding points at the selected locations.

Following completion of the alignment process, the gap distance between the inner surface of the molding plates and their corresponding cast was measured virtually. Measurements were performed at identical predefined points for all plates, thereby enabling uniform comparison across materials, as shown in Figure 2.

### 2.6. Measurement of Retentive Force

The retention of the PSIO plates was evaluated using a cast model fabricated from soft gum material and printed from the same STL file as the working cast. Retentive force was measured with a traction device equipped with a digital force gauge (500 N, Xmoonfree Digital Force Gauge, Forest Hills, NY, USA), offering a resolution of 0.1 N and an accuracy of 0.5%. The printed soft cast was securely fixed to the stand of the traction device with the palatal surface oriented upwards. To simulate intraoral conditions, an aqueous 80% glycerol solution was applied as a lubricant to the cast.

A commercially available denture adhesive paste (Polygrip Zinc-free Denture Adhesive Cream; Haleon, Ireland) was applied between the maxillary model and the feeding plate. A plastic syringe was used to ensure consistent and standardized application of the adhesive. Controlled pressure of 10–12 N was applied to the molding plate for 20 s to achieve uniform distribution of the adhesive across the entire mucosal surface. 

Following pressurization, traction force was applied to the plate from the position corresponding to its center of gravity, directed perpendicularly to the imaginary occlusal plane. The point at which the plate detached from the cast was recorded as the retentive value, representing the maximum traction force. Each measurement was repeated three times per plate, and the mean of these values was calculated and recorded as the retentive force. For each repetition, the plate was completely removed, and the adhesive was freshly reapplied, thereby avoiding changes in adhesive behavior or surface conditions. Statistical analyses were subsequently performed using the mean value per plate, thereby yielding 10 experimental units for each material group. This procedure ensured that repeated measurements from the same plate were appropriately consolidated and not treated as independent observations, thus preserving the validity of the statistical comparisons.

### 2.7. Statistical Analysis 

Data were processed using SPSS Statistics version 25 (IBM, Armonk, NY, USA). The assumptions of normality and homogeneity were assessed with Shapiro–Wilk and Levene’s tests. Descriptive statistics, including means, standard deviations (SDs), and 95% confidence intervals (CIs), were computed. Because the assumption of homogeneity of variances was violated, Welch’s ANOVA was employed, followed by post hoc testing to identify significant differences across regions and materials. The threshold for statistical significance was set at α = 0.05, with α = 0.01 indicating the highest level of significance.

#### Reliability Analysis

Intraclass correlation coefficients (ICCs) were computed to assess intra-examiner reliability for measurement accuracy. Ten samples were measured twice, with a one-week interval between sessions. The analysis demonstrated strong agreement under both the ‘Single’ and ‘Average’ conditions (ICC = 0.992a, 0.997c), confirming consistent and dependable ratings within a narrow confidence interval. For the retention test, both conditions also showed moderate-to-high agreement (ICC = 0.930a, 0.962c) across repeated measurements. Overall, these findings indicate that measurements obtained from the same subjects were stable and reproducible.

## 3. Results

### 3.1. Overall Surface Adaptation

The distance between the inner surface of the plates and their corresponding casts was measured at 36 standardized points, and the mean of these measurements was calculated to represent the adaptation value of each plate for subsequent statistical analysis. Plates fabricated from PETG/TPU exhibited a mean fitting value of 0.321 ± 0.134 mm, while those made from PETG demonstrated the best accuracy with the smallest gap discrepancy (0.226 ± 0.060 mm). In contrast, direct-printed plates showed the largest mean distance of 0.386 ± 0.120 mm (Table 2). The values were normally distributed, but variance homogeneity was violated; therefore, Welch’s ANOVA was applied instead of the conventional one-way ANOVA. Welch’s test confirmed significant differences among the groups (*p* = 0.005). Post hoc Games–Howell comparisons revealed that PETG plates differed significantly from direct-printed plates (*p* = 0.006), whereas no significant differences were observed between PETG/TPU and either PETG (*p* = 0.148) or direct-printed plates (*p* = 0.499) (Table 3).

**Figure 3:**
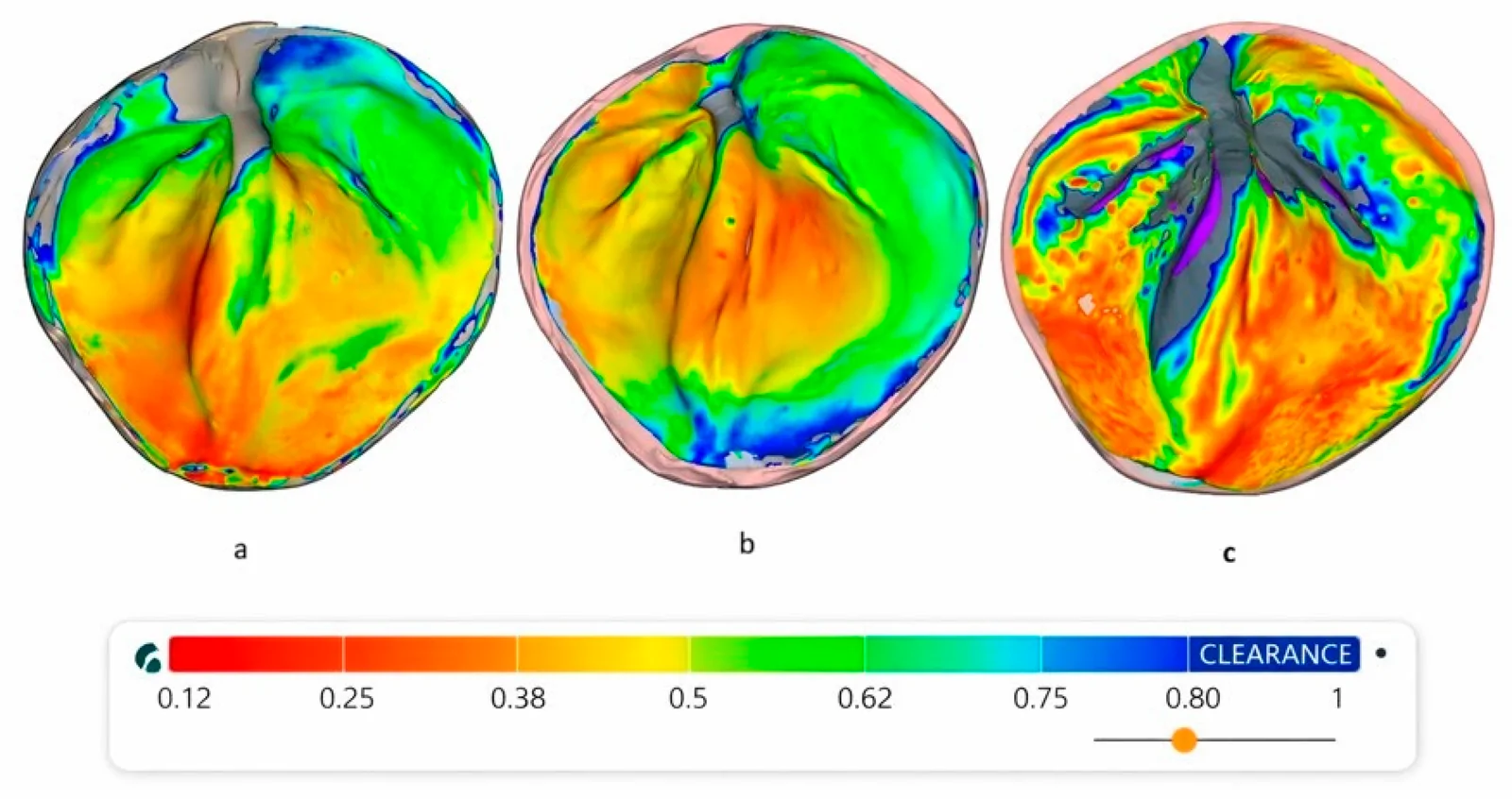
Comparison of the profiles between models and their experimental molding plates fabricated from (**a**) PETG/TPU, (**b**) PETG, and (**c**) direct-printed resin. The color bar represents the distance in mm.

Figure 3 demonstrates a color map comparison of surface adaptation among experimental molding plates fabricated from three materials. The color scale represents the fitness of the plates in terms of the gap between the plate and the 3D-printed cleft model. Lower values at the red end of the scale (≈0.12) correspond to minimal gap and excellent adaptation, whereas higher values toward the blue end (≈1.0) indicate increased distance between the plate and the model, reflecting poor adaptation. Intermediate colors such as orange and yellow denote moderate gaps, indicative of acceptable adaptation. In contrast, the appearance of grey tones signifies extremely large gaps, representing very poor adaptation beyond the measurable range of the standard scale. This gradation provides a quantitative and visual measure of plate adaptation across the materials. Plates fabricated from PETG showed the most favorable and consistent adaptation among the tested materials (b), particularly along the alveolar ridge regions, represented by red–orange zones, with only minor pressure areas at the central palate (red). Plates produced from the PETG/TPU combination demonstrated intermediate adaptation, with scattered green and blue regions indicating outward deviations in millimeters (a). In contrast, direct-printed plates exhibited the least favorable adaptation profile, characterized by extensive green and blue zones (c). Most notably, these plates revealed a larger gap represented in the grey zone, highlighting their limited accuracy compared to the other materials.

### 3.2. Region-Specific Misfit

Across all three materials, the greatest degree of misfit was consistently observed in the anterior regions. Among them, PETG molding plates demonstrated the most favorable adaptation across all regions, outperforming the other materials in terms of overall fit (Table 4). Based on the Games–Howell multiple comparisons, the results show clear differences in misfit across the three materials. In the anterior region, PETG/TPU hybrid plates and direct-printed plates exhibited significantly greater misfit compared to PETG plates, confirming PETG as the most stable material anteriorly (Table 5). No statistically significant differences in misfit levels were observed among the three materials in either the middle or posterior regions, indicating comparable performance. Overall, PETG plates consistently showed the lowest misfit in the anterior region, while PETG/TPU and direct-printed plates displayed higher misfit, but differences diminished in the middle and posterior regions, leading to similar performance across all materials in the middle and posterior regions.

### 3.3. Retention

In the present study, a one-way ANOVA was conducted to compare mean values among the material groups. The assumptions of normality and homogeneity of variances were confirmed prior to analysis. Given that variances were equal between groups, Tukey’s HSD post hoc test was applied to identify specific pairwise differences while maintaining control over Type I error. PETG exhibited the highest mean retentive force (4.30), which was significantly greater than both PETG/TPU (2.280) and direct-printed plates (0.93). Additionally, PETG/TPU showed significantly higher retentive force compared to direct-printed plates, as shown in Table 4. These results highlight clear material-dependent differences in retention, with PETG demonstrating superior performance, followed by PETG/TPU, while direct-printed plates exhibited the lowest retentive capacity. One-way ANOVA and post hoc testing (Tukey’s HSD) revealed a highly significant difference between PETG and the two other materials (Table 6).

## 4. Discussion

Retention is a critical determinant of the clinical performance of removable intraoral appliances, as it directly influences appliance stability, patient comfort, and treatment effectiveness [31,32,33]. The accurate adaptation and secure retention of presurgical infant orthopedic (PSIO) appliances are essential for successful cleft management, as they facilitate alveolar segment alignment, improve feeding efficiency, and enhance overall treatment outcomes and caregiver satisfaction [24,34,35]. In conventional removable prostheses, retention is achieved through multiple mechanisms, including mechanical engagement of tooth undercuts, muscular control, implant-supported attachment systems, and the posterior palatal seal, which enhances stability by creating a peripheral seal between the hard and soft palate [36]. Although plate thickness and fabrication conditions were standardized, plate weight was not directly measured. Minor variations in mass between materials could have influenced the measured retentive force, since differences in density may alter stress distribution during dislodgement [37]. 

The effectiveness of these mechanisms depends on the appliance design, material properties, and the accuracy of tissue adaptation [36,37]. In contrast, retention of presurgical infant orthopedic (PSIO) plates in infants with cleft palate is considerably more challenging because the cleft disrupts the continuity of the palatal vault, preventing the formation of an effective palatal seal [38,39]. Consequently, PSIO appliances rely primarily on intimate adaptation to the available mucosal tissues, engagement of anatomical undercuts, and, when necessary, the use of auxiliary retention methods such as denture adhesives [24,40,41]. Because denture adhesive was applied uniformly across all specimens, it represents an integral component of the experimental retention system. Adhesive use enhances stability and simulates clinical practice, but it also contributes to the measured retention force beyond the intrinsic properties of the plate materials [24]. Thus, the observed differences should be interpreted as the combined effect of material characteristics and adhesive-mediated bonding. Importantly, since adhesive application was standardized, relative comparisons between groups remain valid. Among the evaluated materials, PETG provided the most favorable combination of fitness and stability, suggesting that its material properties are better suited to maintaining intimate tissue contact and consistent appliance retention. These findings support the use of materials that optimize both mechanical performance and patient comfort while reducing dependence on conventional acrylic resins and auxiliary retention methods, such as denture adhesives [42,43].

Previous investigations have primarily evaluated the accuracy and clinical performance of materials used for splints [44,45], palatal plates [41,46], and removable dental appliances [47], whereas comparative evidence regarding contemporary materials specifically developed for PSIO appliances remains limited. The present study addresses this gap by directly comparing thermoformed PETG, PETG/TPU hybrid, and directly printed Tera Harz resin under standardized conditions. The findings demonstrated that PETG achieved favorable adaptation accuracy and retention, whereas the PETG/TPU hybrid exhibited intermediate performance and directly printed resin showed the least favorable outcomes. These results indicate that although emerging digital materials expand the available manufacturing options for PSIO appliances, their clinical performance depends not only on fabrication technology but also on the intrinsic properties of the material. Consequently, standardized evaluation of both adaptation and retention is essential before introducing new materials into routine clinical practice. These findings suggest that both the manufacturing technique and the intrinsic properties of the material substantially influence the dimensional fidelity and clinical performance of PSIO appliances. While comparative evidence regarding these contemporary materials remains limited, the present results provide novel data indicating the favorable performance of thermoformed PETG in digital PSIO fabrication. This observation is particularly relevant in light of recent advances in clear aligner materials and digital manufacturing technologies, where multilayer polymers such as PETG and TPU, together with CAD/CAM and additive manufacturing, have expanded the range of materials available for orthodontic applications [48]. The dimensional fidelity of PSIO appliances is a key determinant of their ability to achieve intimate adaptation to the oral tissues, thereby enhancing appliance stability and retention during clinical use [24,49]. 

In the present study, directly printed plates exhibited the lowest adaptation accuracy and retentive force, which may be attributed to several manufacturing- and material-related factors. Polymerization shrinkage, warpage, and dimensional changes during printing and post-curing can compromise the conformity of directly printed appliances to the underlying anatomy [50]. Furthermore, the inherent layer-by-layer fabrication process produces surface irregularities that limit intimate tissue adaptation compared with thermoformed PETG appliances [51,52]. The mechanical characteristics of printable resins, including their lower dimensional stability and greater susceptibility to deformation under functional loading, may further contribute to the reduced fit and retention observed in this study [53].

## 5. Limitations

This study has several limitations. It was limited to an in vitro evaluation of complete unilateral cleft lip and palate (CL/P) models and did not include other cleft types or long-term clinical validation. The in vitro design does not replicate the complex intraoral environment, and the use of a single patient-derived digital cleft model restricts anatomical variability and may introduce dependence among plates fabricated on the same model. The relatively small sample size further limits generalizability. Moreover, the application of denture adhesive during retention testing, while necessary for standardization, may have influenced the results. The absence of saliva, temperature, moisture, soft-tissue compliance, and infant-related functional forces reduces the clinical relevance of the findings. Finally, the lack of long-term and clinical validation underscores the need for future investigations incorporating larger, anatomically diverse samples under dynamic intraoral conditions. Plate thickness and fabrication conditions were standardized across groups; however, plate weight was not directly measured, and minor variations in mass cannot be entirely excluded as a potential influence on retentive force. Following consultation of the EQUATOR Network, no specific reporting guideline was directly applicable to this in vitro experimental design; nevertheless, general principles of methodological transparency and reproducibility were adopted, and this limitation has been acknowledged.

## 6. Clinical Relevance and Future Directions

The present findings demonstrate that material selection significantly influences the adaptation accuracy and retention of digitally fabricated PSIO appliances, with PETG showing the most favorable overall performance. These results support the use of PETG as a promising material for digital PSIO fabrication in unilateral cleft lip and palate. Future longitudinal clinical studies involving different cleft phenotypes are required to validate these findings and evaluate the long-term clinical performance of digitally fabricated appliances.

## 7. Conclusions

This in vitro study demonstrated that material selection influences the adaptation accuracy and retention of digitally fabricated presurgical infant orthopedic (PSIO) plates. Among the materials evaluated, thermoformed PETG exhibited comparatively better fit and retentive force under the tested conditions, suggesting its potential suitability for digitally fabricated PSIO appliances. PETG/TPU showed intermediate performance, whereas directly printed Tera Harz resin demonstrated lower adaptation accuracy and retention. These findings provide preliminary evidence supporting PETG as a promising material for improved appliance stability and adaptation in unilateral cleft lip and palate models. However, given the in vitro nature of this investigation and the absence of clinical or longitudinal validation, the results should be interpreted cautiously. Further clinical studies with larger sample sizes and long-term follow-up are required to confirm the translational relevance of these findings.

## Figures and Tables

**Table 1:** The materials used in the study.

Item	Manufacture
Thermoformed aligner PETG/TPU	Erkodent, Pfalzgrafenweiler, Germany
Thermoformed aligner PETG	Erkodent, Pfalzgrafenweiler, Germany
Direct-printed aligner Tera Harz TC-85	Graphy Inc., Seoul, Republic of Korea
Fast-set addition polyvinylsiloxane impression material	Prestige, Florence, Italy
Type IV stone	Zhermack, Badia Polesine, Italy
Glycerin aqueous solution (80%)	MP Bio medicals, Santa Ana, CA, USA
Resin for 3D printing dental models, dental gum resin	Asiga, Sydney, NSW, Australia
Titanium dioxide spray	Dentaco, Essen, Germany

**Table 2:** Values of accuracy data for the overall area of each type of molding plate.

Descriptive Values	Accuracy		
	PETG/TPU	PETG	Direct-Printed
Mean	0.321	0.226	0.386
St. Deviation	0.134	0.060	0.120
St. Error	0.042	0.019	0.038
Minimum	0.143	0.188	0.168
Maximum	0.510	0.366	0.531
Shapiro–Wilk value	0.907	0.674	0.940
Levene’s value	0.03	0.03	0.03
95% CI (Lower–Upper)	0.225–0.417	0.183–0.270	0.300–0.473

**Table 3:** Welch and Games–Howell values relating to accuracy.

*p*-Value from Welch’s Test		0.005
*p*-value from Games–Howell		
PETG	PETG/TPU	0.148
	Direct-Printed	0.006
PETG/TPU	Direct-Printed	0.499

**Table 4:** Descriptive characteristics were obtained during the accuracy and retention tests.

Descriptive Values	Accuracy									Retention		
	PETG/TPU			PETG			Direct-Printed			PETG/TPU	PETG	Direct-Printed
	Ant.	Mid.	Post.	Ant.	Mid	Post.	Ant.	Mid.	Post.			
Mean	0.475	0.258	0.237	0.191	0.181	0.305	0.511	0.270	0.324	2.280	4.30	0.93
St. Deviation	0.244	0.095	0.089	0.100	0.054	0.070	0.11	0.145	0.208	0.688	0.398	0.246
St. Error	0.077	0.03	0.028	0.031	0.017	0.022	0.034	0.046	0.065	0.217	0.126	0.077
Minimum	0.223	0.134	0.073	0.074	0.092	0.207	0.345	0.076	0.082	1.650	3.65	0.55
Maximum	0.836	0.411	0.303	0.357	0.302	0.438	0.685	0.483	0.685	4.1	4.9	1.3
Shapiro–Wilk value	0.835	0.886	0.720	0.896	0.852	0.938	0.958	0.936	0.841	0.717	0.961	0.963
95% CI (Lower–Upper)	0.301–0.650	0.191–0.327	0.173–0.301	0.120–0.264	0.143–0.220	0.255–0.356	0.312–0.474	0.166–0.375	0.175–0.474	1.79–2.77	4.02–4.59	0.75–1.11

**Table 5:** Post hoc analysis of the anterior, middle, and posterior regions (Games–Howell values).

Material		*p* Values of Games–Howell		
		Anterior	Middle	Posterior
PETG	PETG/TPU	0.014	0.100	0.168
	Direct-Printed	<0.001	0.211	0.960
PETG/TPU	Direct-Printed	0.905	0.975	0.466

**Table 6:** ANOVA and Tukey’s HSD test data for the retention test.

Material		ANOVA	Tukey’s HSD
PETG	PETG/TPU	<0.001	<0.001
	Direct-printed		<0.001
PETG/TPU	Direct-printed	<0.001	<0.001

## Data Availability

The original contributions presented in this study are included in the article. Further inquiries can be directed to the corresponding author.
